# Use of paediatric face mask for adult ventilation in a patient with nasal tumour

**DOI:** 10.4103/0019-5049.60513

**Published:** 2010

**Authors:** Sameer Sethi, Vikramjeet Arora, Hemant Bhagat, Arun Sharma

**Affiliations:** Postgraduate Institute of Medical Education and Research, Chandigarh, India

Sir,

While managing difficult airway, the anaesthesiologist must be able to assess and anticipate the degree of difficulty and then select the method most likely to succeed. Mask ventilation is an essential component of airway management and the delivery of general anaesthesia. Successful mask ventilation provides anaesthesiologists with a rescue technique during unsuccessful attempts at laryngoscopy and unanticipated difficult airway situations. We are reporting a case of a big nasal tumour with impossible conventional mask ventilation successfully managed by ventilation with paediatric face mask covering the mouth only.

**Figure 1:**
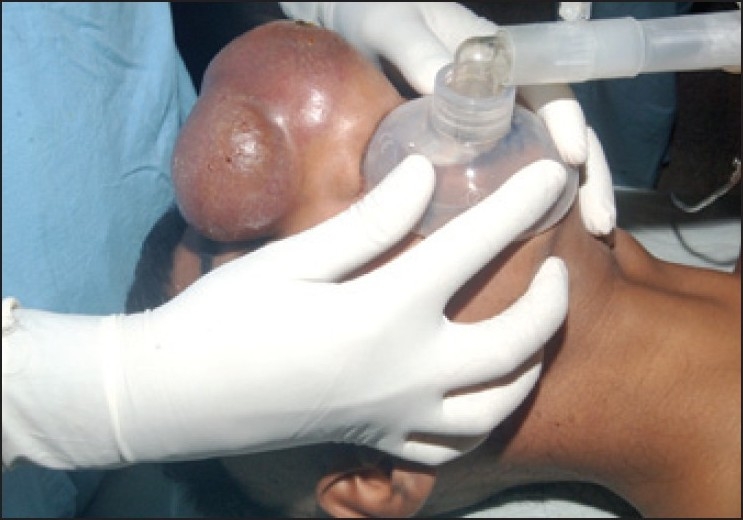
Ventilation through small -sized mask

A 14-year-old male was admitted to the ENT department with a large, round cell tumour of the nose. The patient was posted for excision of mass after routine laboratory investigations. During pre-anaesthetic checkup, we noted that the tumour was covering the whole nose, and the anterior nares were compressed. Other than the mass, airway assessment was unremarkable. No difficulty in intubation was anticipated. The possibility of tracheostomy was mentioned and consent taken. Difficult airway cart was kept ready. Because of the size of the tumour, it was not possible to ensure good facemask seal for initial ventilation. We thought of ventilating the patient through mouth only using paediatric-sized face mask (No. 2) [Figure 1]. After pre-oxygenation, we induced the patient with fentanyl and propofol. Check ventilation was done, and it was adequate through the small-sized mask. Intubation was done with succinylcholine. Rest of the period was uneventful. The patient was extubated on table after the procedure.

Nasal tumour is one of the conditions associated with difficult or impossible mask ventilation. Airway management in a patient with nasal tumour is very challenging because of the inability to place a face mask. In some of these patients, however, tracheal intubation may be achieved easily. An alternative approach in this setting may be the use of laryngeal mask airway. Shimosaka *et al.*[1] reported the use of intubating laryngeal mask airway to maintain spontaneous respiration, followed by fibreoptic bronchoscopy for intubation, in a similar case. The frequency of inability to ventilate and intubate has been estimated at 0.01 to 2.0 per 10,000 anaesthetic administrations[2]; but with proper assessment and planning, such a situation can be prevented. In this particular case, we knew that correct conventional mask ventilation will not be possible, so we decided to use smaller mask covering the mouth only. Nagaro *et al.*[3] used paediatric mouth mask for fibreoptic nasal tracheal intubation in anaesthetised patients to prevent reduction in manoeuvrability of fiberoptic bronchoscope and endotracheal tube as seen while using conventional mask and diapharam for fiberoptic intubation. In this, ventilation and anaesthesia are maintained by an infant- or child-type seal mask applied only over the mouth with the aid of an oral airway during fibreoptic tracheal intubation, and fibreoptic nasal tracheal intubation is performed by another anaesthetist. Thus such types of cases can be managed in centres where fibreoptic bronchoscope is not available.

If there are high chances that both ventilation and intubation will be difficult, airway should be secured while the patient is still awake. In managing difficult airway, proper assessment, preparation and well-organized approach will further bring down airway-related morbidity and mortality.
